# Prevalence of hepatitis B and C viruses in HIV-positive patients in China: a cross-sectional study

**DOI:** 10.7448/IAS.19.1.20659

**Published:** 2016-03-14

**Authors:** Jing Xie, Yang Han, Zhifeng Qiu, Yijia Li, Yanling Li, Xiaojing Song, Huanling Wang, Chloe L Thio, Taisheng Li

**Affiliations:** 1 Department of Infectious Diseases, Peking Union Medical College Hospital, Peking Union Medical College and Chinese Academy of Medical Sciences, Beijing, China; 2 Division of Infectious Diseases, Johns Hopkins Hospital, Johns Hopkins University School of Medicine Baltimore, MA, USA

**Keywords:** HIV, hepatitis B virus, hepatitis C virus, co-infection, prevalence, liver disease, CD4+T cell count

## Abstract

**Introduction:**

Liver disease related to hepatitis B (HBV) and hepatitis C (HCV) may temper the success of antiretroviral therapy (ART) in China. Limited data exist on their prevalence in HIV-positive Chinese. A multi-centre, cross-sectional study was carried out to determine the prevalence and disease characteristics of HBV and HCV co-infection in HIV-positive patients across 12 provinces.

**Methods:**

HIV-positive ART-naïve patients were recruited from two parent cohorts established during November 2008–January 2010 and August 2012–September 2014. Hepatitis B surface antigen (HBsAg), hepatitis B e antigen and HCV antibody (anti-HCV) status were retrieved from parent databases at the visit prior to ART initiation. HBV DNA was then determined in HBsAg+ patients. HCV RNA was quantified in anti-HCV+ patients. Aspartate aminotransferase-to-platelet ratio index (APRI) and the fibrosis-4 (FIB4) were calculated. Chi-square test, Kruskal–Wallis test and logistic regression were used for statistical analysis, as appropriate.

**Results:**

Of 1944 HIV-positive patients, 186 (9.5%) were HIV–HBV co-infected and 161 (8.3%) were HIV–HCV co-infected. The highest HIV–HBV prevalence (14.5%) was in Eastern China while the highest HIV–HCV prevalence was in the Central region (28.2%). HIV–HBV patients had lower median CD4 + T cell count (205 cells/μL) than either HIV monoinfected (242 cells/μL, *P*=0.01) or HIV–HCV patients (274 cells/μL, *P*=0.001). Moderate-to-significant liver disease was present in >65% of the HIV–HCV, ~35% of the HIV–HBV and ~20% of the HIV monoinfected patients. Independent associations with moderate-to-significant liver disease based on APRI included HBV (Odds ratio, OR 2.37, *P <* 0.001), HCV (OR 9.64, *P<*0.001), CD4 count≤200 cells/μL (OR 2.55, *P<*0.001) and age ≥30 years (OR 1.80, *P*=0.001).

**Conclusions:**

HBV and HCV prevalence is high in HIV-positive Chinese and differs by geographic region. HBV and HCV co-infection and HIV monoinfection are risks for moderate-to-significant liver disease. Only HIV–HBV is associated with greater HIV-related immunosuppression. Incorporating screening and management of hepatitis virus infections into Chinese HIV programmes is needed.

## Introduction

In the era of antiretroviral therapy (ART), liver disease from hepatitis virus co-infection is a leading cause of morbidity and mortality in the HIV-positive population in North America and Europe [1, 2]. Although hepatitis virus–HIV co-infection has been extensively studied in developed countries [1, 3–5], it has not been well characterized in Asia and Africa where these viruses are highly endemic [6–8]. There is concern that hepatitis virus–related liver disease may threaten the success of ART programmes in developing countries; therefore, understanding the prevalence and disease characteristics of HBV and HCV co-infection with HIV in Asia and Africa is essential.

China has a heavy disease burden of both chronic viral hepatitis infections and HIV/AIDS. In the general Chinese population, the prevalence of hepatitis B surface antigen (HBsAg) in those aged 1–59 years is 7.2% [9] while the estimated HCV prevalence is 1.0–2.9% [6, 7, 10]. However, the prevalence of these viruses in the 780,000 people with HIV/AIDS in China is not known. A National Free Antiretroviral Treatment Program (NFATP) was initiated in 2002. By 2013, 227,489 patients were receiving ART through the programme [11]. However, the HBV and HCV status were unknown in >50% of the patients starting ART between 2010 and 2011 in NFATP [12]. Previous studies have only been conducted at a single site or focused on certain populations, so they do not provide information on the profile of hepatitis virus–HIV co-infection across China [13–15]. Limited multi-centre studies showed that HBsAg seropositivity ranged from 8.7 to 12.5% while seroprevalence of HCV antibody (anti-HCV) varied from 12.2 to 41.8% [12, 16, 17]. However, these studies did not discuss the HIV or hepatitis disease characteristics.

To determine the nationwide prevalence and disease characteristics of HBV and HCV co-infection in HIV patients, a multi-centre study was carried out using patients enrolled in various China AIDS Clinical Trials in 12 provinces.

## Methods

### Study design and participants

This was a cross-sectional study that included ART-naïve participants enrolled in one of the following parent studies: China AIDS Clinical Trial (CACT) 0810 or CACT1215 (ClinicalTrials.gov identifier: NCT00872417 and NCT01844297). For this study, only study entry data prior to ART were included. Participants in CACT0810 were enrolled between November 2008 and January 2010 (*n*=879). As previously described [18], inclusion criteria in the cohort were: age: 18–65 years, CD4+ T cell count <350 cells/μL and ART-naïve. Exclusion criteria included: acute HIV infection, currently active AIDS-defining illness, pregnancy or breastfeeding and active intravenous drug use (IDU). Participants in CACT1215 were enrolled between August 2012 and September 2014 (*n*=1191). CACT1215 had the same inclusion criteria as CACT0810, except for the CD4+ T cell count threshold of 500 cells/μL. The Institutional Review Board of Peking Union Medical College Hospital (PUMCH) approved the parent studies and each participant provided written informed consent. All the data and specimens used in the present study were retrieved from parent databases before ART initiation.

**Figure 1:**
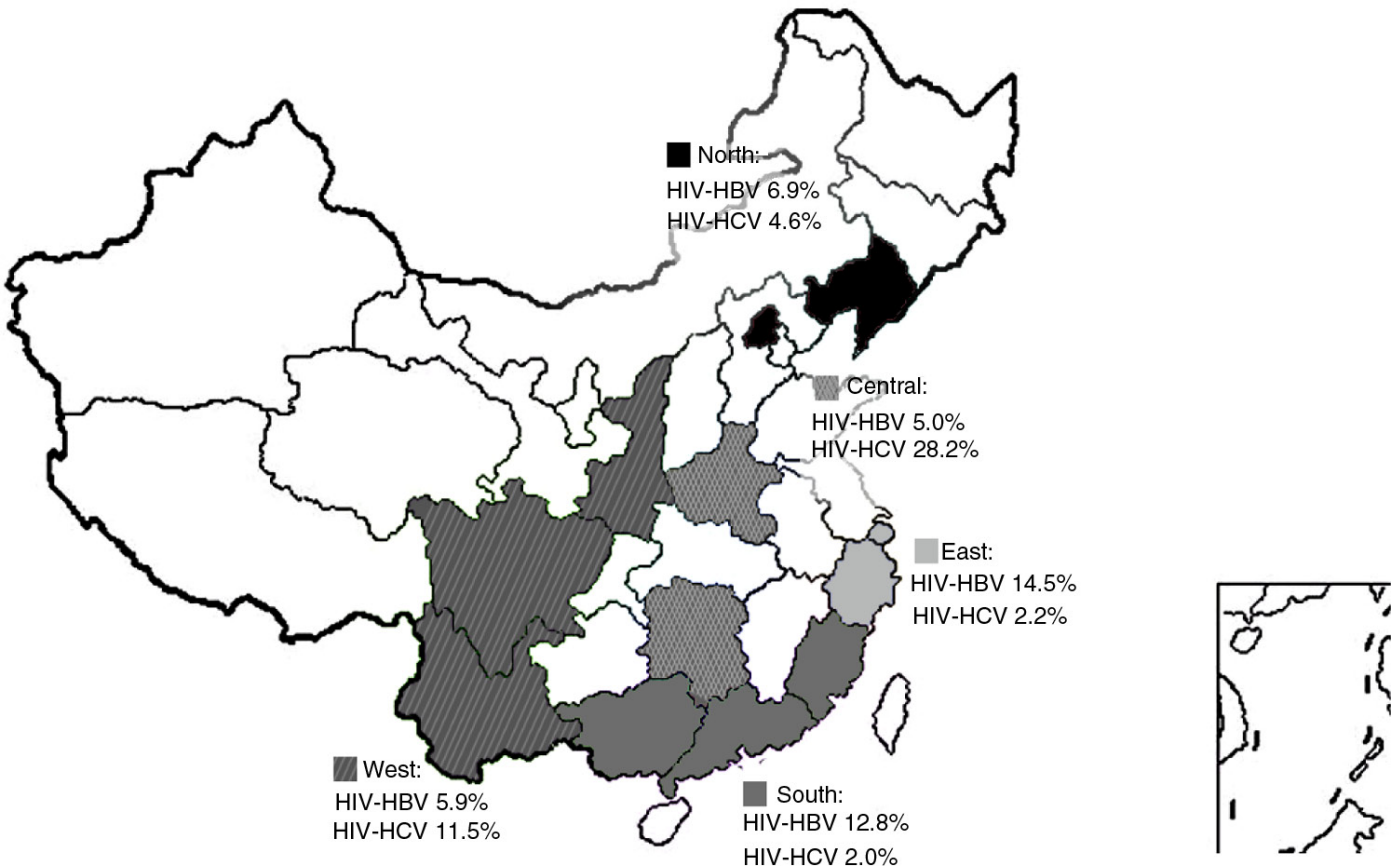
Prevalence of HBV and HCV co-infection in HIV-positive patients by geographic region. Chinese regions as defined in this study are grey or hash-coded. Data indicate estimated prevalence of HBV and HCV co-infection in HIV-positive patients.

Study participants were from one of twelve provinces or municipalities across China, which were clustered into five geographic regions (Figure 1): North, Beijing and Liaoning; East, Shanghai and Zhejiang; South, Guangdong, Guangxi and Fujian; Central, Henan and Hunan; West, Shaanxi, Sichuan and Yunnan. Demographic and clinical data before ART initiation, including age, sex, HIV transmission route, alanine transaminase (ALT), aspartate aminotransferase (AST), platelet count, total bilirubin (Tbil), CD4 + T cell count, HIV RNA, HBsAg, hepatitis B e antigen (HBeAg) and anti-HCV serostatus were abstracted from the parent databases. HBV and HCV serology tests were performed using various commercial-based kits approved by the China Food and Drug Administration. The present study was approved by the Institutional Review Board of PUMCH.

In HBsAg+ patients, HBV DNA was determined. HCV RNA was quantified in anti-HCV+ patients. These values were determined prior to ART initiation. Participants were defined as having HIV–HBV co-infection if they were HBsAg+anti-HCV− or HBsAg+ anti-HCV+HCV RNA−. Participants negative for HBsAg and positive for both anti-HCV and HCV RNA were defined as HIV–HCV co-infected. Participants positive for HBsAg, anti-HCV and HCV RNA were grouped as triply infected. Participants were identified as HIV monoinfected when they were negative for both HBsAg and anti-HCV or HCV RNA.

### Laboratory testing

Plasma HIV RNA, HBV DNA and HCV RNA levels were quantified by the COBAS AmpliPrep/TaqMan48 real-time PCR system (Roche Molecular Systems, Pleasanton, CA, USA) in the Central Laboratory at PUMCH. Plasma samples were separated from whole blood by centrifugation within 4 h of collection and stored at −80°C until tested. The linear range of HIV RNA, HBV DNA and HCV RNA were 40–1,000,000 copies/mL (1.60–6.00 log_10_ copies/mL), 20–170,000,000 IU/mL (1.30–8.23 log_10_ IU/mL) and 15–100,000,000 IU/mL (1.18–8.0 log_10_ IU/mL), respectively.

Two non-invasive markers, AST-to-platelet ratio index (APRI) [19] and the fibrosis-4 score (FIB4) [20], were used to evaluate the liver fibrosis of study participants. APRI was calculated according to Wai *et al*. [19]: (actual AST value divided by its upper normal limit considered as 40 U/L)/platelet counts (10^9^/L)×100. FIB4 score was calculated according to Sterling *et al*. [20]: [age (years)×AST (U/L)]/[platelets (10^9^/L)×(ALT U/L)^1/2^].

### Statistical analysis

ALT and AST values were graded according to the AIDS Clinical Trial Group (ACTG) criteria: normal, values at the upper limits of normal (ULN) (40 U/L); mild, 1.25–2.5×the ULN; moderate, 2.5–5×the ULN; severe, 5–10×the ULN; life-threatening, >10×the ULN [21]. APRI and FIB4 scores were ranked into three classes in accordance with previous studies conducted in HIV-positive population: APRI class 1,≤0.5; APRI class 2, from 0.51 to 1.5; APRI class 3, >1.5; FIB4 class 1, ≤1.45; FIB4 class 2, from 1.46 to 3.25; FIB4 class 3, >3.25 [22].

Comparisons among groups were made by non-parametric methods. Chi-square or Fisher's exact test was used for categorical variables and Kruskal–Wallis test or Mann–Whitney U test was used for continuous variables. Estimated prevalence and 95% confidence interval (CI) were calculated by modified Wald method. Separate univariate binary logistic regression models were used to assess the odds ratio (OR) for elevated liver fibrosis scores with the following cutoffs: APRI > 0.5 and FIB4 > 1.45 (elevated scores). Parameters included in the models were age, sex, CD4+ T cell count, HIV RNA levels and hepatitis virus co-infection status. Multivariable logistic regression models were then developed using covariates that were significant (*P<*0.05) in the univariate models. The analysis was performed among the entire cohort and then restricted to the HIV monoinfected group to identify predictors for increased liver fibrosis scores. Statistical analysis was performed using SPSS 22.0 (IBM Corporation, Armonk, New York, USA) and GraphPad Prism 6.0 (GraphPad Software, Inc. La Jolla, CA, USA). *P* value <0.05 was considered statistically significant.

## Results

### Demographics of participants and prevalence of HBV and HCV in HIV-positive patients

A total of 2070 treatment-naive participants were eligible for this analysis, 74 (3.6%) of whom were excluded because of missing HBsAg and/or anti-HCV results. A further 48 (2.4%) anti-HCV+ patients were excluded because specimens were not available for HCV RNA testing leaving 1948 included in this study. The excluded participants were similar in age, CD4 count and sex to the included participants (data not shown). Of the 1948 participants, 186 (9.5%) were HIV–HBV co-infected, 161 (8.3%) were HIV–HCV co-infected with a mean HCV RNA level of 6.34±1.0 log_10_ IU/mL and 4 (0.2%) had triple infection. Triply infected participants were not included in further analyses because of the small number of subjects.

The median age of the included 1944 participants was 36 years with no differences among the groups (Table 1). HIV–HBV co-infected participants had higher proportion of males (80.6%) than HIV monoinfected (65.4%, *P<*0.001) or HCV-co-infected participants (67.1%, *P=*0.005). In the HIV monoinfected group, heterosexual transmission was most common (61.4%), followed by male-to-male transmission (22.4%). The HIV–HBV co-infected participants had a similar transmission distribution; however, the HIV–HCV co-infected participants had significantly higher proportion of blood-borne transmission (55.9%) than either monoinfection (5.7%, *P<*0.001) or HIV–HBV co-infection (2.7%, *P<*0.001).

**Table 1:** Demographics of participants

	Overall	HIV Monoinfection	HIV–HBV Co-infection	P*	HIV–HCV Co-infection	P*	*P#*
Number	1944	1597	186		161		
Median Age (IQR) (years)	36 (29–45)	36 (29–45)	36 (31–45)	0.44	38 (32–43)	0.14	0.40
Male sex	1302 (67.0%)	1044 (65.4%)	150 (80.6%)	<0.001	108 (67.1%)	0.73	0.005
Transmission				0.29		<0.001	<0.001
Male-to-male	402 (20.7%)	358 (22.4%)	42 (22.6%)		2 (1.2%)		
Heterosexual	1128 (58.0%)	981 (61.4%)	118 (63.4%)		29 (18.0%)		
Bisexual	30 (1.5%)	24 (1.5%)	6 (3.2%)		0 (0.0%)		
Blood transfusion	186 (9.6%)	91 (5.7%)	5 (2.7%)		90 (55.9%)		
Others	46 (2.4%)	11 (0.7%)	1 (0.5%)		34 (21.1%)		
Unknown	152 (7.8%)	132(8.3%)	14 (7.5%)		6 (3.7%)		

* *P* values are for comparisons with HIV monoinfection group.

# *P* values are for comparisons between HIV–HBV and HIV–HCV co-infected groups. HBV=hepatitis B virus; HCV=hepatitis C virus; HIV=human immunodeficiency virus; IQR=interquartile range.

Interestingly, the prevalence of HIV–HBV and HIV–HCV co-infection varied widely by region (Figure 1 and Table 2). Participants in Eastern China had the highest prevalence of HIV–HBV co-infection (14.5%), while the Central region had the lowest of 5.0%. In contrast, the Central region had the highest HIV–HCV prevalence (28.2%) followed by the West (11.5%), the North (4.6%), the East (2.2%) and finally the Southern region (2.0%). In the Central region, 50.2% of participants were infected by blood transfusion, which was significantly higher than that in the other four regions ranging from 11.5% in the North to 1.5% in the South.

**Table 2:** Prevalence of HBV and HCV co-infection in HIV-positive patients by geographic region

	Overall	HIV Monoinfection	HIV–HBV Co-infection		HIV–HCV Co-infection	
			
Region	*N*	*N*	*N*	Estimated prevalence (%) (95% CI)	*N*	Estimated prevalence (%) (95% CI)
Overall	1944	1597	186	9.5 (8.3–10.9)	161	8.3 (7.1–9.6)
North	174	154	12	6.9 (3.6–11.7)	8	4.6 (2.0–8.9)
East	185	154	27	14.5 (9.8–20.4)	4	2.2 (0.6–5.4)
South	806	687	103	12.8 (10.6–15.3)	16	2.0 (1.1–3.2)
Central	257	171	13	5.0 (2.7–8.4)	73	28.2 (22.8–34.1)
West	522	431	31	5.9 (4.1–8.3)	60	11.5 (8.9–14.5)

CI = confidence interval; HBV = hepatitis B virus; HCV = hepatitis C virus; HIV = human immunodeficiency virus.

### HIV disease characteristics

The HIV–HBV co-infected participants had the lowest median CD4+ T cell count (205 cells/μL) compared with the HIV monoinfected (242 cells/μL, *P=*0.01) or the HIV–HCV co-infected (274 cells/μL, *P=*0.001) participants (Table 3). Nearly half of HIV–HBV co-infected participants (47.9%) had CD4+ T cell count below 200 cells/μL compared with 39.7% of the HIV monoinfected group and 36.7% of the HIV–HCV co-infected group. However, the HIV RNA levels did not differ among the groups.

**Table 3:** HIV and liver disease characteristics of study participants by HBV and HCV co-infection status

	HIV Monoinfection	HIV–HBV Co-infection	P*	HIV–HCV Co-infection	P*	*P#*
Median CD4 count (IQR) (cells/μL)	242 (144–334)	205 (122–309)	0.01	274 (148–354)	0.02	0.001
CD4 count			0.15		0.005	0.005
≤100 cells/μL	274 (17.2%)	37 (19.9%)		13 (8.1%)		
101–200 cells/μL	359 (22.5%)	52 (28.0%)		46 (28.6%)		
201–350 cells/μL	646 (40.5%)	69 (37.1%)		62 (38.5%)		
> 350 cells/μL	318 (19.9%)	28 (15.1%)		40 (24.8%)		
Median HIV RNA (IQR) (log_10_ copies/mL)	4.71 (4.30–5.16)	4.69 (4.23–5.16)	0.66	4.60 (4.13–5.05)	0.07	0.23
Median ALT (IQR) (U/L)	22 (16–33)	30 (21–44)	<0.001	45 (27–72)	<0.001	<0.001
ALT grade			0.04		<0.001	<0.001
Normal	1436 (90.1%)	157 (84.4%)		93 (57.8%)		
Mild	141 (8.8%)	25 (13.4%)		52 (32.3%)		
Moderate	17 (1.1%)	4 (2.2%)		12 (7.5%)		
Severe	0 (0.0%)	0 (0.0%)		4 (2.5%)		
Median AST (IQR) (U/L)	24 (20–31)	28 (23–39)	<0.001	46 (33–69)	<0.001	<0.001
AST grade			0.001		<0.001	<0.001
Normal	1003 (95.2%)	113 (86.9%)		67 (55.8%)		
Mild	49 (4.6%)	17 (13.1%)		39 (32.5%)		
Moderate	2 (0.2%)	0 (0.0%)		11 (9.2%)		
Severe	0 (0.0%)	0 (0.0%)		3 (2.5%)		
Median Platelet (IQR) (10^9^/L)	194 (158–232)	174 (137–217)	<0.001	158 (118–200)	<0.001	0.009
Median Tbil (IQR) (mg/dL)	10.5 (7.8–13.9)	11.1 (8.6–15.2)	0.003	12.6 **(**9.7–17.1)	<0.001	0.06
Median APRI (IQR)	0.32 (0.25–0.46)	0.40 (0.30–0.62)	<0.001	0.80 (0.45–1.27)	<0.001	<0.001
APRI			<0.001		<0.001	<0.001
Class 1: ≤0.50	833 (79.3%)	82 (63.1%)		34 (28.3%)		
Class 2: 0.51–1.50	211 (20.1%)	46 (35.4%)		65 (54.2%)		
Class 3: >1.50	7 (0.7%)	2 (1.5%)		21 (17.5%)		
Median FIB4 (IQR)	0.97 (0.70–1.36)	1.03 (0.77–1.62)	0.006	1.76 (1.21–2.51)	<0.001	<0.001
FIB4			0.005		<0.001	<0.001
Class 1: ≤1.45	812 (78.5%)	85 (66.4%)		34 (34.7%)		
Class 2: 1.46–3.25	203 (19.6%)	37 (28.9%)		47 (48.0%)		
Class 3: >3.25	20 (1.9%)	6 (4.7%)		17 (17.3%)		

* *P* values are for comparisons with HIV monoinfection group.

# *P* values are for comparisons between HIV–HBV and HIV–HCV co-infected groups. ALT=alanine transaminase; APRI=AST-to-platelet ratio index; AST=aspartate aminotransferase; FIB4=fibrosis-4; HBV=hepatitis B virus; HCV=hepatitis C virus; HIV=human immunodeficiency virus; IQR=interquartile range; Tbil=total bilirubin.

### Liver disease characteristics

Hepatitis virus co-infected participants had higher median ALT and AST values than HIV monoinfected participants (Table 3). Notably, the highest ALT and AST values were seen in the HIV–HCV co-infected participants with ~10% having moderate or severe elevations. The HIV–HCV co-infected participants also had the highest median APRI score (0.80), followed by HIV–HBV co-infected (0.40, *P<*0.001) and HIV monoinfected participants (0.32, *P<*0.001) (Table 3). Nearly three-quarters of the HIV–HCV co-infected participants had APRI scores >0.50, which is considered as moderate-to-significant hepatic fibrosis. This proportion was much higher than participants with HIV–HBV co-infection (36.9%, *P*<0.001) or HIV monoinfection (20.8%, *P*<0.001). Similar results were seen with FIB4 (Table 3). Multivariable analysis demonstrated that HCV co-infection was the strongest predictor for moderate-to-significant liver disease by both APRI (OR 9.64, 95% CI 5.65–16.45) and FIB4 (OR 5.94, 95% CI 3.48–10.15) (Table 4). Other independently associated variables included HBV co-infection (OR 2.37 for APRI, OR 1.91 for FIB4), CD4 count below 200 cells/μL (OR 2.55 for APRI, OR 2.28 for FIB4) and age ≥30 years (OR 1.80 for APRI, OR 8.81 for FIB4).

**Table 4:** Factors associated with elevated APRI (>0.50) and FIB4 (>1.45) scores in the entire cohort and HIV monoinfected participants

	APRI				FIB4			
		
	Univariate		Multivariable		Univariate		Multivariable	
				
	OR (95% CI)	*P*	OR (95% CI)	*P*	OR (95% CI)	*P*	OR (95% CI)	*P*
Entire cohort								
Age (years)								
< 30	1		1		1		1	
≥ 30	2.00 (1.49–2.69)	<0.001	1.80 (1.29–2.51)	0.001	9.36(5.96–14.71)	<0.001	8.81 (5.42–14.31)	<0.001
Male sex								
No	1				1			
Yes	1.05 (0.81–1.38)	0.70			0.81 (0.62–1.06)	0.12		
CD4 count (cells/μL)								
> 350	1		1		1		1	
350–201	1.26 (0.87–1.83)	0.22	1.60 (0.96–2.64)	0.07	1.78 (1.18–2.70)	<0.001	1.89 (1.13–3.15)	0.02
≤200	1.96 (1.36–2.82)	<0.001	2.55 (1.55–4.20)	<0.001	2.50 (1.66–3.78)	<0.001	2.28 (1.37–3.81)	0.002
HIV RNA ≥ 5.16 log_10_ copies/mL (the upper quartile)								
No	1		1		1		1	
Yes	1.49 (1.09–2.04)	0.01	1.33 (0.94–1.87)	0.11	1.67 (1.22–2.27)	0.001	1.36 (0.96–1.92)	0.08
Hepatitis virus co-infection								
No	1		1		1		1	
HBV	2.24 (1.52–3.29)	<0.001	2.37 (1.57–3.59)	<0.001	1.84 (1.24–2.74)	0.002	1.91 (1.23–2.95)	0.004
HCV	9.67 (6.32–14.77)	<0.001	9.64 (5.65–16.45)	<0.001	6.85 (4.41–10.66)	<0.001	5.94 (3.48–10.15)	<0.001
HIV monoinfected group								
Age (years)								
< 30	1		1		1		1	
≥ 30	1.71 (1.22–2.42)	0.002	1.67 (1.15–2.43)	0.008	9.64 (5.61–16.58)	<0.001	9.50 (5.28–17.03)	<0.001
Male sex								
No	1				1		1	
Yes	0.95 (0.69–1.31)	0.74			0.68 (0.49–0.92)	0.01	0.87 (0.61–1.23)	0.42
CD4 count (cells/μL)								
> 350	1		1		1		1	
350–201	1.49 (0.92–2.42)	0.10	1.82 (1.02–3.25)	0.04	1.93 (1.16–3.21)	0.01	1.98 (1.11–3.52)	0.02
≤200	2.48 (1.54–3.98)	<0.001	2.85 (1.61–5.05)	<0.001	3.06 (1.85–5.06)	<0.001	2.61 (1.47–4.64)	0.001
HIV RNA ≥ 5.16 log_10_ copies/mL (the upper quartile)								
No	1		1		1		1	
Yes	1.79 (1.24–2.57)	0.002	1.42 (0.97–2.08)	0.08	1.96 (1.38–2.79)	<0.001	1.41 (0.96–2.07)	0.08

APRI=AST-to-platelet ratio index; CI=confidence interval; FIB4=fibrosis-4; HBV=hepatitis B virus; HCV=hepatitis C virus; HIV=human immunodeficiency virus; OR=odds ratio.

In the subset of HIV monoinfected subjects (Table 4), CD4 count below 350 cells/μL was associated with moderate-to-significant liver disease by both APRI (OR 1.82, 95% CI 1.02–3.25) and FIB4 (OR 1.98, 95% CI 1.11–3.52) scores. CD4 count≤200 cells/μL was even more strongly associated (Table 4). Age≥30 years was significantly associated with elevated APRI (OR 1.67, 95% CI 1.15–2.43) and FIB4 (OR 9.50, 95% CI 5.28–17.03) scores.

In the 186 HIV–HBV co-infected participants, 57 (30.6%) were positive for HBeAg. Age, sex and distribution of HIV transmission routes did not differ between participants with and without HBeAg (Table 5). HBeAg-positive patients trended towards lower CD4+ T cell counts (median 188 cells/μL, IQR 106–283 cells/μL) than HBeAg-negative participants (median 214 cells/μL, IQR 132–312 cells/μL, *P*=0.21). No difference was found in HIV RNA levels between the two subgroups. Plasma to measure HBV DNA was available in 43 (75.4%) HBeAg-positive and 105 (81.4%) HBeAg-negative participants. The median HBV DNA was 8.03 log_10_ IU/mL in the HBeAg-positive participants, which was higher than in the HBeAg-negative participants (3.02 log_10_ IU/mL, *P*<0.001). HBeAg-positive participants had higher median ALT than HBeAg-negative participants (36 U/L vs. 27 U/L, *P*<0.001), but other markers of liver disease did not differ between the two groups (Table 5).

**Table 5:** Characteristics of HIV–HBV co-infected participants by HBeAg status

	HBeAg+	HBeAg−	P*
Number (%)	57 (30.6%)	129 (69.4%)	
Age (IQR) (years)	35 (30–46)	36 (31–45)	0.57
Male sex	48 (84.2%)	102 (79.1%)	0.55
Transmission			0.40
Male-to-male	13 (22.8%)	29 (22.5%)	
Heterosexual	36 (63.2%)	82 (63.6%)	
Bisexual	1 (1.8%)	5 (3.9%)	
Blood transfusion	0 (0.0%)	5 (3.9%)	
Others	0 (0.0%)	1 (0.8%)	
Unknown	7 (12.3%)	7 (5.4%)	
Median CD4 count (IQR) (cells/μL)	188 (106–283)	214 (132–312)	0.21
CD4 count			0.66
≤100 cells/μL	12 (21.1%)	25 (19.4%)	
101–200 cells/μL	19 (33.3%)	33 (25.6%)	
201–350 cells/μL	19 (33.3%)	50 (38.8%)	
> 350 cells/μL	7 (12.3%)	21 (16.3%)	
Median HIV RNA (IQR) (log_10_ copies/mL)	4.77 (4.42–5.33)	4.63 (4.19–5.14)	0.10
Median HBV DNA (IQR) (log_10_ IU/mL)	8.03 (6.93–8.23)	3.02 (1.91–4.73)	<0.001
HBV DNA			<0.001
Undetectable (<20 IU/mL)	4 (9.3%)	20 (19.0%)	
20–2000 IU/mL	1 (2.3%)	41 (39.0%)	
2001–20,000 IU/mL	0 (0.0%)	15 (14.3%)	
20,001–200,000 IU/mL	1 (2.3%)	7 (6.7%)	
> 200,000 IU/mL	37 (86.0%)	22 (21.0%)	
Median ALT (IQR) (U/L)	36 (29–50)	27 (19–40)	<0.001
ALT grade			0.009
Normal	44 (77.2%)	113 (87.6%)	
Mild	9 (15.8%)	16 (12.4%)	
Moderate	4 (7.0%)	0 (0.0%)	
Median AST (IQR) (U/L)	33 (24–43)	28 (23–36)	0.09
AST grade			0.39
Normal	32 (82.1%)	81 (89.0%)	
Mild	7 (17.9%)	10 (11.0%)	
Median platelet count (IQR) (10^9^/L)	171 (124–212)	176 (141–222)	0.37
Median Tbil (IQR) (mg/dL)	11.2 (9.3–15.7)	11.0 (8.4–15.0)	0.32
Median APRI (IQR)	0.40 (0.30–0.67)	0.39 (0.31–0.60)	0.54
Median FIB4 (IQR)	1.00 (0.65–1.70)	1.04 (0.84–1.59)	0.65

* *P* values are for comparisons between HBeAg-negative and HBeAg-positive groups.

ALT=alanine transaminase; APRI=AST-to-platelet ratio index; AST=aspartate aminotransferase; FIB4=fibrosis-4; HBV=hepatitis B virus; HCV=hepatitis C virus; HIV=human immunodeficiency virus; IQR=interquartile range; Tbil=total bilirubin.

## Discussion

In this large study of HIV-positive persons across China, HBV and HCV co-infection was found in 9.5 and 8.3% of subjects, respectively. Notably, the distribution of co-infection was not uniform across the country and was different between HBV and HCV. We found that the HIV–HBV co-infected participants were more immunosuppressed than HIV monoinfected or HIV–HCV co-infected participants as demonstrated by having the lowest CD4+ T cell counts. A large proportion of all subjects had moderate-to-significant liver disease as determined by the serum markers APRI and FIB4 with the greatest proportion in HIV–HCV co-infected participants (>65%). CD4+ T cell count <200 cells/μL was an independent risk factor for elevated liver fibrosis scores regardless of hepatitis status. Taken together, these data demonstrate that HIV monoinfected persons can have moderate-to-significant liver disease and that co-infection with these hepatitis viruses is common in HIV-positive Chinese and leads to important clinical consequences, which are currently unrecognized.

The prevalence of HIV–HBV co-infection in our study is consistent with previous multi-centre studies conducted in China ranging from 8.7 to 12.5% [12, 16, 17]. In accord with a previous multinational study [3], HIV–HBV co-infected participants had lower CD4+ T cell counts than HIV monoinfected participants. In contrast, the retrospective study with data from the China NFATP demonstrated that CD4+ T cell counts did not differ between HIV–HBV co-infection and HIV monoinfection [12]. One possible reason is that the NFATP study had more participants with CD4+ T cell counts below 200 cells/μL and had a lower overall median CD4+ T cell count making it more difficult to find a difference in CD4+ T cell counts between groups.

Of the 186 HIV–HBV co-infected patients, 30.6% were positive for HBeAg, which is markedly lower than previous studies from the United States (59%) [23], Canada (54%) [4] and the multinational study (50%) [3]. This difference may be due to differences in age of HBV acquisition. In countries such as the United States and Canada with low endemicity, HBV acquisition primarily occurs in adulthood. In contrast, the major mode of HBV acquisition in China is mother-to-child [24]; thus, they have been infected with HBV for years and so are more likely to have HBeAg-negative disease [25]. The Chinese national surveys indicate that the HBeAg prevalence inversely correlates with age and that the HBeAg prevalence in that survey for the age of our participants is similar to our findings [9, 24]. Regardless of HBeAg status, over one-third of the HIV–HBV co-infected patients had moderate-to-significant liver disease, so diagnosing and treating HBV is imperative as HBV treatment can improve liver disease [26, 27].

The anti-HCV seropositivity rate (14.0%) in our cohort is within the range of 12.2 to 41.8% as seen in previous Chinese multi-centre studies [12, 16, 17]. However, our results, for the first time, demonstrate the prevalence of HCV co-infection of 8.3% since we tested HCV RNA in anti-HCV positive patients. Identifying patients with HIV–HCV co-infection is important since we found that over two-thirds had moderate-to-significant liver disease as measured by either APRI or FIB4. By non-invasive measurements, the prevalence of fibrosis in previous studies of HIV–HCV co-infected patients is around 30–50% in other countries [20, 28, 29]. One possible explanation for the higher prevalence of moderate-to-significant liver disease in our cohort is that our participants had lower median CD4+ T cell counts, which we found to be an independent risk factor for elevated APRI and FIB-4. The NFATP report showed that HCV co-infection is associated with higher mortality in the two-year period after ART initiation compared with those infected by HIV alone [12]. Taken together with our study, HCV co-infection may have clinical consequences in HIV-positive individuals on ART supporting the need for testing and treatment for HCV in ART treatment programmes in China. Even if HCV treatment is not readily available across China, these patients could be prioritized for HIV treatment regardless of CD4 cell count because some studies have shown that liver disease improves with ART [30, 31].

The other notable finding in our study is that about 20% of the HIV monoinfected individuals had moderate-to-significant liver disease. This is consistent with other studies from the United States but higher than in an international cohort [32, 33]. We also found that liver disease in this group was strongly associated with CD4+ T cell count≤350 cells/μL using APRI and FIB4, as was demonstrated in a US-based study [32, 33]. These data suggest that immunosuppression from HIV affects liver disease and supports earlier treatment of HIV disease. It is not clear why HIV monoinfected individuals have significant liver disease, but it may be related to HIV infection of hepatic stellate cells or hepatocytes, microbial translocation or an inflammatory state in the liver from HIV infection [34–36]. Further work is also needed to determine whether ART improves liver disease in the HIV monoinfected individuals.

This study has several limitations. First, patients were recruited from previous ART trials; thus, we cannot rule out that there were differences between patients who were included in parent trials and those not included. Second, active IDUs were not included in parent studies; thus, our results cannot be generalized to HIV-positive IDUs in China. However, since the current HIV epidemic is via sexual transmission [11], our results are timely. Third, inclusion criteria of parent studies may exclude patients with very high ALT or AST values, so we may have underestimated the number of co-infected people with advanced liver disease. Fourth, we could not collect data on other potential liver disease–related factors such as alcohol use and metabolic syndrome. Regarding the difference of liver fibrosis in HIV monoinfected patients between this cohort and international studies, these hepatic comorbid factors should be taken into account. Finally, we did not have liver biopsy data and instead used APRI and FIB4 to evaluate liver fibrosis. Although these markers correlate well with liver disease in other countries, they have not been extensively validated in China. However, it is likely that they are representative of liver disease stage based on other studies.

## Conclusions

This study is the first, to our knowledge, to characterize HBV and HCV co-infection in treatment-naïve HIV-positive patients in China. Our data demonstrate that there is a high prevalence of HBV and HCV co-infection and that co-infection is associated with important medical consequences. As ART scale up increases through China's treatment programme, liver disease from hepatitis virus co-infection and even from HIV monoinfection will be an emerging problem. Thus, incorporation of screening and effective treatment of hepatitis virus infection into general HIV management in China is imperative.

## Competing interests

The authors declare no competing interests.

## Authors' contributions

TL, CLT and JX were responsible for study design, whereas JX, YH, ZQ, YL, YL, XS and HW collected the data. Data analysis was done by JX, while JX, CLT and TL drafted the manuscript. All authors have critically read the manuscript.
